# Implementing a simplified neonatal resuscitation protocol-helping babies breathe at birth (HBB) - at a tertiary level hospital in Nepal for an increased perinatal survival

**DOI:** 10.1186/1471-2431-12-159

**Published:** 2012-10-05

**Authors:** Ashish KC, Mats Målqvist, Johan Wrammert, Sheela Verma, Dhan Raj Aryal, Robert Clark, Naresh P KC, Ravi Vitrakoti, Kedar Baral, Uwe Ewald

**Affiliations:** 1 International Maternal and Child Health, Department of Women’s and Children’s Health, Uppsala University, Uppsala, Sweden; 2 Population Service International, Kathmandu, Nepal; 3 Paropakar Maternity and Women’s Hospital, Kathmandu, Nepal; 4 Latter Day Saints Charity, Salt Lake City, USA; 5 Ministry of Health and Population, Kathmandu, Nepal; 6 Patan Academy of Health Sciences, Patan, Nepal

## Abstract

**Background:**

Reducing neonatal death has been an emerging challenge in low and middle income countries in the past decade. The development of the low cost interventions and their effective delivery are needed to reduce deaths from birth asphyxia. This study will assess the impact of a simplified neonatal resuscitation protocol provided by Helping Babies Breathe (HBB) at a tertiary hospital in Nepal. Perinatal outcomes and performance of skilled birth attendants on management of intrapartum-related neonatal hypoxia will be the main measurements.

**Methods/Design:**

The study will be carried out at a tertiary level maternity hospital in Nepal. A prospective cohort-study will include a six-month baseline a six month intervention period and a three-month post intervention period. A quality improvement process cycle will introduce the neonatal resuscitation protocol. A surveillance system, including CCD cameras and pulse oximeters, will be set up to evaluate the intervention.

**Discussion:**

Along with a technique to improve health workers performance on the protocol, the study will generate evidence on the research gap on the effectiveness of the simplified neonatal resuscitation protocol on intrapartum outcome and early neonatal survival. This will generate a global interest and inform policymaking in relation to delivery care in all income settings.

**Trial registration:**

ISRCTN97846009

## Background

The Millennium Development Goals 4 and 5, aimed respectively at reducing child mortality and improving maternal health, [1], are inextricably linked [2,3]. Improvements in maternal health will lead to reduction in intrapartum and newborn deaths. However, the progress towards reaching the goals by 2015 remains unsatisfactory since of the 68 Countdown countries only 19 are on track to achieve MDG 4 [4]. Though there has been significant progress in reducing the under-five mortality in the last decade, the rate of reduction in neonatal death has been relatively slow, with still more than 40% of child deaths occurring in the neonatal period [5,6]. One fourth of the death during the first four weeks of life is attributed to intrapartum-related hypoxia, “birth asphyxia”, also known as intrapartum related death [5,7,8].

Nearly all (99%) intrapartum-related deaths occur in low and middle income countries with 60 million births each year outside health facilities and up to one-half without a skilled birth attendant [9,10]. Every year, approximately 904,000 intrapartum-related neonatal deaths and 1.02 million intrapartum stillbirths [11] occur globally, with intrapartum-related conditions making up for 23% of neonatal deaths and 32% of stillbirths [12]. Among the survivors of intrapartum-related hypoxia “birth asphyxia”, one million may develop cerebral palsy, learning difficulties, or other forms of disability each year [13].

There has been growing evidence that a simple intervention can save a large number of babies from intrapartum hypoxia. Among the 10-20% of babies who do not breathe at birth, many respond to drying, warming, clearing of the airways, and specific stimulations. Only a small number (an estimated 3-6%) require bag-and-mask ventilation, and less than 1% require advance methods of resuscitation, such as chest compressions and medications [14].

Even though some proven interventions exist to prevent intrapartum and early neonatal death, health workers often fail to perform according to established guidelines [15,16]. The poor performance of health worker has multiple determinants ranging from proximal ones such as a lack of knowledge, skill and motivation to distal factors such as disabling working environment in health facilities (poor clinical practices, leadership and supervision; lack of adequate supplies and equipment; health workers’ ineffective participation in planning; and lack of peer support). Understanding the critical determinants of performance and accordingly developing specific interventions to improve is a key to achieve quality delivery of health services [17]. Systematic review by the Effective Practice and Organization of Care (EPOC) Cochrane group showed that multi-faceted interventions - training and supervision - improve health workers’ performance [17-19]. Furthermore, a randomized controlled trial of training on neonatal resuscitation in health facilities and community showed a reduction in stillbirths, suggesting an increased recognition of babies who are not breathing, but who can respond to simple measures [12]. An analysis of seven facility-based studies estimated that a neonatal resuscitation educational intervention reduced the neonatal mortality rate from 17% to 43% [12,14,20-25].

### Helping Babies Breathe (HBB) at birth

As a response to reduce the burden of intrapartum deaths and let health workers implement effective resuscitation practices, the American Academy of Pediatrics has developed a simplified neonatal resuscitation protocol: Helping Babies Breathe (HBB). Designed as an educational program in resource limited settings, HBB seeks to prepare birth attendants to care for healthy newborns and those who are not breathing at birth. Ideally, at every birth there should be a skilled person who can provide essential services to both mother and infant, including HBB. HBB is focused on the first minute of birth, also called Golden Minute®, when either stimulating or ventilating with bag-and-mask can save a life [14].

### Context of perinatal and child survival in Nepal

In Nepal, there have been remarkable reductions in both under-five child mortality and maternal mortality over the last 15 years. However, the reduction in neonatal and perinatal mortalities has been relatively slow. According to the Nepal Demographic Health Survey 2011, neonatal mortality contributes 61% of all the under-five deaths. Even though the coverage of skilled birth attendance at birth has doubled in the last five years, the neonatal mortality has stagnated within the same period [26].

To mitigate the neonatal death from three of the main causes - infection, preterm birth complication and birth asphyxia - Ministry of Health and Population developed a National Neonatal Health Strategy in 2004 [27] to guide interventions at different tiers of the health system. Similarly, the National Safe Motherhood and Neonatal Long Term Plan −2006 [28] seeks to integrate neonatal intervention into maternal health services. Recognizing a growing need of adequate skilled workers who could provide care and support to women from pregnancy to postpartum, MoHP developed the Skilled Birth Attendant Policy [29]. Moreover, a community based newborn care package [30] was developed and scaled up to reduce neonatal death in community setting. However, the neonatal resuscitation protocol has yet to be tested for effectiveness in both the national skilled birth attendant training package as well as the community-based newborn care package.

### Study objective

This study aims to assess the impact of HBB at a tertiary hospital in Nepal on perinatal outcomes and performance of skilled birth attendants on management of intrapartum related neonatal hypoxia.

#### Primary research question

Will HBB improve the intrapartum and early neonatal outcome?

#### Secondary research question

Will a set of multifaceted interventions i.e. and continuous quality assessment and improvement cycle improve the performance of birth attendants in HBB?

Will the educational program of HBB improve the competency and confidence of birth attendants?

## Methods/Design

### Setting

The research will be conducted in Paropakar Maternity and Women’s Hospital (PMWH), a tertiary government hospital providing gynecological and obstetrics services in Kathmandu, Nepal. Equipped with 415 inpatient beds, it serves as the central referral hospital and the training site for reproductive health including neonatal health in the country. Among 641 staff members, it has 70 doctors, 175 nurses, 46 paramedics, 63 administrative workers, and 271 junior staff. The hospital provides specialty services as well as academic programs like postgraduate diploma and Masters in obstetrics and gynecology.

Of the 23,155 total deliveries in the 12 months between 2011–2012, 22,957 were live births. Maternal mortality rate was 17 per 100,000 for the same period. Neonatal admission was in excess of neonatal beds available. Early Neonatal mortality rate was currently at nine per 1000 live births, and stillbirth rate was 19 per 1000 birth. Delivery rate per day was 63. Abnormal deliveries accounted for 31% of the total number of deliveries. The rate of Caesarean Section was 20%.

The hospital has an admission unit, a labor room, a maternal and newborn service center, an operation theatre, an antenatal care unit, a maternal intensive care unit and a postnatal ward for admission, delivery and postnatal care of women and newborns.

### Trial design

A prospective cohort study covering periods both before and after the intervention will be administered. Baseline will be conducted covering a period of six months before the intervention. While the Intervention period will be of six months, a post-intervention evaluation will last for three months.

There will be a case-referent design interviewing all mothers who had a newborn requiring resuscitation (cases) as well as a random selection of 20% of all pregnant women admitted to PMWH (referents).

### Intervention

**Figure 1:**
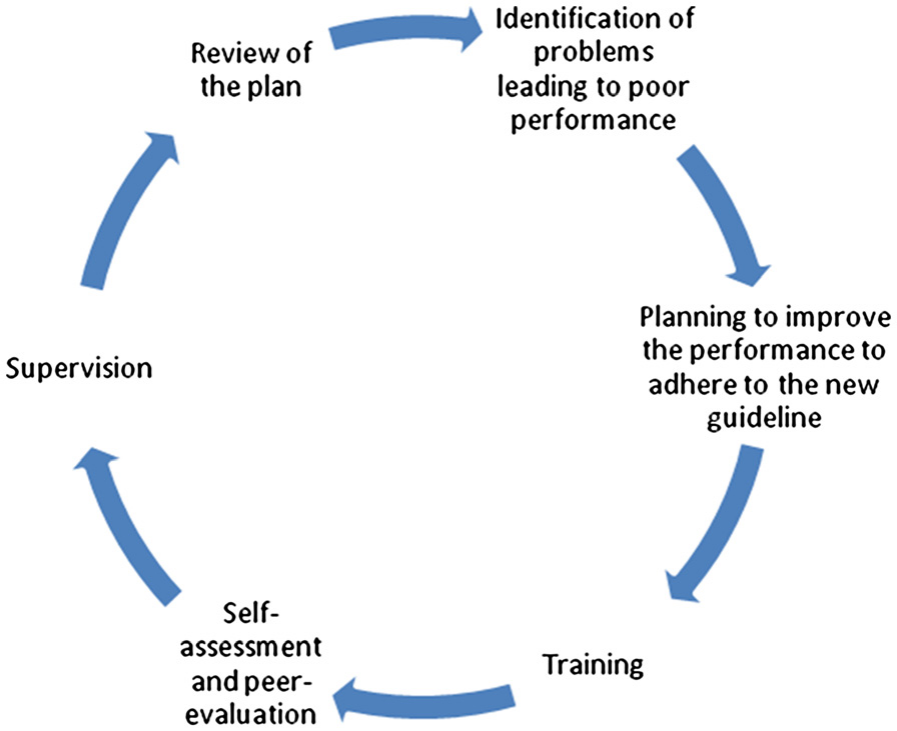
**Quality improvement cycle [**[17]**,**[31]**].**

After the baseline period, a simplified algorithm for neonatal resuscitation at birth - HBB- will be implemented in the hospital. In order to ensure the birth attendants adhere to the new guideline on neonatal resuscitation, a framework of quality improvement cycle will be introduced. This quality improvement cycle will assess the critical determinants of performance and introduce a system of continuous quality assessment and improvement process (Figure 1). The different parts of this quality improvement cycle are:

1. *Identification of problem* - The performance of the birth attendants on neonatal resuscitation will be discussed in groups. A facilitator, selected among the birth attendants, will review and compare the performance against the baseline data.

2. *Planning* - Based on the problems identified, the birth attendants will plan and implement the simplified neonatal resuscitation guidelines using the continuous improvement cycle-training, self-assessment, peer-evaluation, supervision and review. This planning will determine the performance as well as measure the changes in performance on periodic basis.

3. *Training* - Based on the planning, birth attendants will be introduced to HBB techniques using educational materials. Training of Trainers (TOT) will be provided to the in-charge and the facilitator of the unit equipping them with the techniques to recuperate babies who are not breathing at birth. The TOT will also impart the pedagogic skills as per the HBB protocol.

The trained birth attendants will then train other staffs in the birthing center, labor room, admission room and operation theatre in batches of three. The pre-service students who come for placements in labour room will also be trained by the trained birth attendant and supervised by facilitator. So, there will be a three generation of training.

4. *Self assessment and peer evaluation* - The trained birth attendant will perform equipment checks and runs a drill of HBB in Neonatalie using a checklist. In the event health workers have dealt with birth asphyxia, the managing team would conduct a group evaluation in a white-board-checklist, while the health worker would self-evaluate in paper. In the delivery logbook, the health worker will sign against the post-resuscitation procedure column.

5. *Supervision* - The in-charge will ensure whether the equipment and drill of HBB checklist has been filled as required on a daily basis and provide feedback to those who have not. The in-charge will check the delivery logbook to ensure that the case of birth asphyxia is recorded correctly and that the post-resuscitation procedure is signed off. The in-charge will provide timely feedbacks based on findings and ensure that the non-functioning equipment are replaced.

6. *Review* - The staffs of each unit will meet once a month to review the progress of the quality improvement plan and discuss the changes in neonatal resuscitation as well as the factors determining the performance.

### Data collection and outcome

Data will be collected on three main domains: the intervention process, healthcare process indicators, and the primary outcomes-perinatal mortality (Table 1) of the intervention.

**Table 1:** Outcomes and methods of data collection for evaluation of the introduction of a simplified resuscitation protocol (Helping Babies Breathe) in a tertiary hospital in Kathmandu, Nepal

	**Variables**	**Methods of data collection**
**Intervention process**	▪ Knowledge level and in-training performance of health staff; knowledge of perinatal care practices, performance of resuscitation algorithm on Neonatalie®	▪ Pre-defined in-training observational protocols
	▪ Attitudes and perceptions of perinatal health problems and HBB training.	▪ Knowledge assessment questionnaires
	▪ The factors determining the performance of health worker	▪ Health workers survey
	▪ The facilitative skills of the facilitator and group meeting	▪ Focus group discussions.
		▪ Observation checklist
**Healthcare process data**	▪ Effects of resuscitation; APGAR score [32-37] at 1, 5 and 10 minutes, saturation at 5 and 10 minutes (all newborns requiring resuscitation)	▪ Surveillance
	▪ Risk factors for intra-partum related death; socioeconomic status, obstetric history, antenatal care attendance etc. (cases and referents)	▪ Case-referent interviews
	▪ Performance of health staff at resuscitation; time to initiation, time to effective ventilation (all resuscitation situations)	▪ CCD camera evaluation
	▪ Health status of baby after discharge	▪ Pulse-oximeter
		▪ Follow up interviews with case and referent
**Primary outcome**	▪ Perinatal mortality	▪ Surveillance

#### The intervention process

The intervention process will be monitored continuously. The questionnaires from the HBB training package will be used to measure knowledge level and in-training performance of the participants. The factors determining the performance of health worker will be measured using quantitative tools such as health workers profile and qualitative tools such as focus group discussions. The facilitating skills of the facilitator and the quality of group meetings will be assessed using observation checklist. Tools to assess the change in group behavior will be introduced.

#### Healthcare process data

A surveillance system for the research project will be established to assess the perinatal outcome, clinical outcome of resuscitation, and clinical performance of health staff in resuscitation.

Budget Dome (CCD) Cameras and pulse oximeters were placed at each resuscitation table. CCD camera is of MTC-505DH model with a progressive scan sensor and excellent low light performance. Wireless Pulse Oximeter is CMS-50E model of Contec Medical System, China which has USB with wireless interface to Computer.

A total of six CCD cameras and six pulse oximeters with high accuracy and capability to store data will be installed. Technical experts will be invited to train the surveillance officers so that data could be retrieved locally. Similarly, birth attendant from different units will be trained by technical experts to ensure they can use the equipment in a resuscitation situation. The CCD cameras will store information on treatment of babies that were unable to breathe at birth. Each surveillance officer in his/her shift will check the functional status of the pulse oximeter and CCD camera. The checklists will ensure the equipment was monitored every day.

Randomization will be conducted to select referent mother using blinded selection process. An opaque jar containing 100 balls will be placed in the admission room, of which eighty balls will be white and 20 will be yellow. When a woman is admitted for delivery, the surveillance officer would take out a ball from the jar. If the ball is yellow, the woman will be the ‘referent’ and accordingly a referent form (pink color) will be attached to the admission record journal. If the ball is white, no such action will follow. Each ball withdrawn from the jar will be kept separate in a new jar. In terms of human resources, a total of 12 surveillance officers with public health backgrounds with a reasonable experience in administering surveys will be posted at admission and postnatal unit. A rotational 24-hour shift will ensure that a surveillance officer is present twenty-four hours in the admission room throughout the study period.

Periodic follow up interviews will be carried out with parents to assess the health outcomes of the babies undergoing treatment.

#### Primary outcome

The primary outcome- perinatal mortality- will be evaluated using surveillance data.

#### Data collection tools

Five tools will be used: four of them record forms; one interview form.

##### Summary record register (S-1)

The summary referent register has been developed to keep key information on referent women. The information include date and time of admission, place of referral, and a summary of discharge.

##### Referent record form (form A)

A randomized sample of 20% women registered at the admission is selected as referents. The referent record form provides previous and current obstetrics history as well as background and nutritional information of referent woman, including current intrapartum and postpartum information.

##### Case record form (form B)

Defined as ‘cases’ are women delivering babies who are not breathing at birth. The case record form captures woman’s background, nutritional information, previous obstetric and current pregnancy history, resuscitation, intrapartum and postpartum information.

##### Interview questionnaire (form C)

Both cases and referents will be interviewed by the surveillance officer to assess the socio-economic, education, exposure to health information, knowledge, perception and practice on care during pregnancy, delivery and postnatal care, birth preparedness and care seeking behavior. The interviews will be conducted before they are discharged.

##### CCD and pulse oximeter observation record form (form D)

All cases of resuscitation are recorded by CCD camera and oxygen saturation levels are measured by oximeters. Information from these tools is recorded in a observation record form. This observation form monitors the performance of birth attendants as well as clinical outcomes of the resuscitation.

### Sample size and target population

The women delivering and the birth attendants working at the site will be the target population. We conservatively estimate that there will be over 23,000 pregnancies per year in the study area. Given the level of current perinatal mortality of 30/1000, the routine reporting system can demonstrate a significant reduction of 7/1000 and bigger, i.e. to 23/1000 or less (alfa 0.05, beta 0.2) over a six month period.

### Timeline

A prospective cohort baseline will capture information on women delivering in the hospital from July-December 2012 (six months). A set of interventions will follow for six months between January to June or August of 2013, followed again by a post intervention evaluation period of three months.

### Data management

The surveillance officer will fill up and assess records and the interview forms followed by a thorough reevaluation by a research coordinator. A data entry officer will re-check them for discrepancies before entering the data in computers. The research coordinator will verify at least 10% of the accuracy in record forms with that from its primary source similar, will also observe the interviews (this sentence need to be rewritten). In order to avoid data loss a protocol for data tracking system will be followed. Similarly, bi-monthly internal and external verifications or audits will be conducted to ensure data completeness and accuracy.

The Census and Survey Processing System (CSPro), a public domain software package developed and supported by the U.S. Census Bureau and ICF Macro, will be used for quality data management. CSPro is interfaced with SPSS (originally, Statistical Package for the Social Sciences), which will be used for statistical analysis, data management (case selection, file reshaping, creating derived data), and data documentation. Hard copies of records will be stored in a filing system in a secure room. Data will be checked for accuracy, consistency, and completeness in both the CSPro and SPSS. An analysis plan will be developed in accordance with the reporting guidelines. A profile and a comparison of key variables between cases and referents at baseline will be presented.

### Interim analysis

We will form a Data Monitoring Committee on June 2012 to be convened on a 6 monthly basis thereafter. The board will include national and international experts for the trial and will review data and procedures according to the DAMOCLES guidelines [38]. Apart from checking the adequacy of enrolment, the committee will not only ensure the study protocol was followed, but also verifies whether the randomization has produced comparable groups so that, in the absence of interventions, it would be reasonable to expect similar health outcomes over time. It will also review interim data for completeness, quality, and adherence to ethical requirements, and accordingly make recommendations on the continuation of the trial or its modifications.

### Ethical issues

The study was approved by the Nepal Health Research Council (Reg. No. 37/2012) and the Ethical Review Board of Uppsala University (dnr 2012/267). There had been several interactive meetings with hospital administration and health workers to conceptualize the research. The discussions were also held with academicians, researcher, policy makers, and representatives of local and international nongovernmental organizations and associations where the plans and objectives of the research were presented. Written consent form has been developed for potential referents to see whether or not they are willing to participate. The study will only work with the respondents after their consents are recorded. The study team will regularly follow up with the babies of the referent and case parents.

### Sustainability and scalability

Aligned with the national needs, this research seeks to identify a cost effective intervention that can be effectively implemented to improve the performance of health workers to reduce intrapartum and neonatal deaths. The intervention is replicable and can be scaled-up by setting up a steering committee- comprising of MoHP, academia, associations, researchers, Nepal Health Research Council, International NGO, and health journalists- that not only guides implementation, but also uses findings to inform policy dialogues. The research will promote public engagement locally, nationally and internationally. The hospital management and health workers will be contacted regularly to share the progress of research and provide feedback on quality of hospital management. The results will be disseminated to hospital and there will be a discussion to identify solutions to sustain potential positive results. Dissemination of the protocol and findings of the trial will be published in national and international peer-reviewed journals.

## Discussion

To provide a scalable strategy to improve the health workers performance in simplified neonatal resuscitation to reduce intrapartum death has been a global research priority [14,17]. In low resource healthcare setting with multiple and complex factors determining the performance of health worker, identifying a specific solution to improve performance would be crucial. The HBB initiative has previously been shown to be feasible and acceptable to health workers [39,40]. The focus on assisted breathing and the “golden minute” harbor an expectation also for improved results in relation to mortality and morbidity. This has however not been demonstrated. The results from this research study will therefore be of importance for policy makers at all levels and have implications for future neonatal resuscitation guidelines globally.

A recent systematic review by Child Health and Nutrition Initiative (CHNRI), WHO, and experts has identified the need of evidence for a simpler and more robust technology for neonatal resuscitation, resuscitation training, as well as more feasible models of maintaining clinical competency for resuscitation as one of the top ten research priorities in order to reduce deaths from birth asphyxia by 2015 [41,42]. There are hospital based studies done in various settings that has shown that neonatal resuscitation can significantly reduce early neonatal mortality as well as intrapartum still birth, but that could not explain the activities performed to improve the performance of health workers [20,22-25]. The “black box” of implementation needs to be further investigated and elements that enhance knowledge translation need to be tested in a scientific way.

The research will find effective and feasible methods of knowledge translation in order to improve newborn health and survival from birth asphyxia which is a global research priority essential for reaching the Millennium Development Goals 4 and 5. We therefore propose an intervention method using a continuous quality improvement cycle. Through this intervention package the process of implementation will be in the hands of the health workers themselves and not dictated from the top. Problems of managing neonatal asphyxia will be identified, planning will be done to improve practice in accordance with the new guideline, and a system of self evaluation, peer evaluation and group review will be put into practice by this bottom-up approach. Unlike the traditional method of imposing new guidelines through training to improve the performance, the foundation of this method lies on realization of existing problems in care provision. This method will not only bring behavior change at individual level but also at organizational level.

The tools to measure clinical performance of the health workers and clinical outcome of the newborns used in this trial are in a sense novel in this setting. By utilizing modern information technology by the application of CCD cameras and pulse-oximeters, we will be able to refine and elaborate our outcome measures. This will achieve an increased understanding of what is going on in the resuscitation situation.

## Competing interests

Laerdal foundation for Acute Medicine had no influence on conceptualization or design of the study and holds no claim to results or intellectual property produced by the study.

## Authors’ contributions

AK, MM, JW and UE conceptualized and designed the study. AK, MM and UE were principal applicants for funding. AK and MM drafted the study protocol manuscript. All authors provided intellectual input and approved the final manuscript.

## Pre-publication history

The pre-publication history for this paper can be accessed here:

http://www.biomedcentral.com/1471-2431/12/159/prepub
